# Prevalence of gastro-oesophageal reflux disease symptoms and reflux-associated respiratory symptoms in asthma

**DOI:** 10.1186/1471-2466-10-49

**Published:** 2010-09-15

**Authors:** Lakmali D Amarasiri, Arunasalam Pathmeswaran, H Janaka de Silva, Channa D Ranasinha

**Affiliations:** 1 Department of Physiology, Faculty of Medicine, University of Kelaniya, Ragama, Sri Lanka; 2 Department of Public Health, Faculty of Medicine, University of Kelaniya, Ragama, Sri Lanka; 3 Department of Medicine, Faculty of Medicine, University of Kelaniya, Ragama, Sri Lanka; 4 Department of Pharmacology, Faculty of Medicine, University of Kelaniya, Ragama, Sri Lanka

## Abstract

**Background:**

Gastro-oesophageal reflux disease (GORD) symptoms are common in asthma and have been extensively studied, but less so in the Asian continent. Reflux-associated respiratory symptoms (RARS) have, in contrast, been little-studied globally. We report the prevalence of GORD symptoms and RARS in adult asthmatics, and their association with asthma severity and medication use.

**Methods:**

A cross-sectional analytical study. A validated interviewer-administered GORD scale was used to assess frequency and severity of seven GORD symptoms. Subjects were consecutive asthmatics attending medical clinics. Controls were matched subjects without respiratory symptoms.

**Results:**

The mean (SD) composite GORD symptom score of asthmatics was significantly higher than controls (21.8 (17.2) versus 12.0 (7.6); *P *< 0.001) as was frequency of each symptom and RARS. Prevalence of GORD symptoms in asthmatics was 59.4% (95% CI, 59.1%-59.6%) versus 28.5% in controls (95% CI, 29.0% - 29.4%). 36% of asthmatics experienced respiratory symptoms in association with both typical and atypical GORD symptoms, compared to 10% of controls (*P *< 0.001). An asthmatic had a 3.5 times higher risk of experiencing a GORD symptom after adjusting for confounders (OR 3.5; 95% CI 2.5-5.3). Severity of asthma had a strong dose-response relationship with GORD symptoms. Asthma medication use did not significantly influence the presence of GORD symptoms.

**Conclusions:**

GORD symptoms and RARS were more prevalent in a cohort of Sri Lankan adult asthmatics compared to non-asthmatics. Increased prevalence of RARS is associated with both typical and atypical symptoms of GORD. Asthma disease and its severity, but not asthma medication, appear to influence presence of GORD symptoms.

## Background

Gastro-oesophageal reflux disease (GORD) symptoms are common in asthmatics. A recent systematic review on GORD symptom assessment in asthmatics reported an estimated prevalence ranging from 30-90% compared to 10-20% in the general population [1]. A study from South East Asia reported a GORD symptom prevalence of 57% in asthmatics compared to 34% in non-asthmatic controls [2], and a study of an urban Indian population found that 74.8% of asthmatics had a history of GORD [3]. There is also evidence that the severity and frequency of GORD symptoms are related to the severity of asthma [4-6]. Individual asthma drugs have also been shown to predispose towards GORD [7-11]. In addition to a higher prevalence of GORD symptoms, asthmatics also associate GORD symptoms with respiratory symptoms, the so-called Reflux Associated Respiratory Symptoms (RARS). Several studies report chronic respiratory manifestations of GORD [12-14]. Others report an association between reflux and cough [15,16] and reflux and bronchial hyperresponsiveness [17-19]. However few studies have investigated whether asthmatics experience respiratory symptoms at the time of GORD symptoms [20,21] and none from the Asian continent.

In this study, we investigated the prevalence of GORD symptoms and RARS in a population of adult asthmatics attending a teaching hospital in Sri Lanka, and the association of asthma severity and asthma medication use with the presence of GORD symptoms.

## Methods

A cross-sectional analytical survey was carried out prospectively at the Colombo North Teaching hospital in Sri Lanka over a two year period. Ethical approval was obtained from the Scientific and Ethics Review Committee of the Faculty of Medicine, University of Kelaniya. Adult asthmatics and matched non-asthmatics between the ages of 15-60 years were recruited from medical clinics in the hospital. Those who had previous oesophageal or gastric disease other than GORD, had undergone previous upper gastro-intestinal surgery or had known diabetes mellitus were excluded. All subjects gave informed written consent before the study.

Consecutive asthmatics either newly diagnosed or in follow-up, were recruited according to American Thoracic Society guidelines for the definition of asthma [22]. Controls were either clinic attendees, those accompanying them or hospital staff who did not have respiratory symptoms, asthma or other respiratory illness.

### Study protocol

#### Diagnosis of asthma

Subjects were asked five questions to assess presence or absence of asthma symptoms, in relation to a recall period of 12 months preceding the interview [23].

(1) Have you at any time had wheezing or whistling in your chest?

(2) Have you at any time been breathless when you had wheezing or whistling in your chest?

(3) Have you at any time been woken up with a feeling of tightness in your chest?

(4) Have you at any time been woken up by an attack of coughing?

(5) Have you at any time been woken up by an attack of shortness of breath?

Those with a positive response to one or more questions underwent a white cell and differential count examination and a plain chest radiograph. In those with absolute eosinophilia, the *Wuchereria bancrofti *antigen test (NOW^® ^Filariasis, rapid ICT, Binax, Scarborough, USA) and examination of stools for helminth ova using the direct smear method [24] were done to exclude alternative diagnoses (tropical pulmonary eosinophilia, Loeffler's syndrome). Spirometry was performed using a portable hand-held spirometer (Micro Plus spirometer, Micro Medical Limited, Rochester, UK). Forced Vital Capacity (FVC), Forced Expired Volume in the first second (FEV_1_), Peak Expiratory Flow Rate (PEFR) were recorded after each of three consecutive forced expiratory efforts, both pre and post bronchodilator administration (15 minutes after inhalation of 400 μg of salbutamol from a metered dose inhaler via a valved spacer). A 12% improvement and 200 mL increase in FEV_1 _following bronchodilator administration was considered diagnostic of asthma. Predicted values for Sri Lankans were derived from data of Udupihille et al. [25]. Body mass index (BMI) was calculated for each subject.

#### Assessment of GORD symptoms

All asthmatics and controls were interviewed by the same investigator using a previously validated GORD scale that assessed the frequency and severity of seven symptoms of reflux disease (table 1) using a recall period of the past 4 weeks. For each symptom the patient was asked two questions: "how often do you have this problem?" to assess frequency, and "how much does this problem bother you?" to assess severity. The frequency and severity were scored on a Likert scale (table 1). The subjects were given a cue card from which they chose the relevant response. A composite symptom score was calculated as the sum of products of symptom frequency and severity of each symptom. This resulted in scores from 1 to 20 for each symptom, with a total maximum score of 140 and a minimum score of 7. A subject was categorized as GORD symptom positive if the composite GORD score cut-off ≥ was 12.5 and as GORD symptom negative if the cut-off was <12.5. A subject categorized as GORD symptom negative experienced either infrequent or no symptoms [26].

**Table 1:** Wording of description of gastro-oesophageal reflux disease (GORD) symptoms

Symptom*	Description
Heartburn	Burning pain in chest or abdomen
Regurgitation	Episodes of bitter or sour fluid or food coming back to your mouth
Abdominal/chest pain	Upper abdominal pain or chest pain other than the burning pain in the chest or abdomen
Abdominal distension	Feeling of fullness in your upper abdomen
Dysphagia	Difficulty in swallowing
Cough	Episodes of cough during the day following a meal or at night or when you wake up
Belching	Episodes of wind coming up into your mouth

*Each symptom was asked in relation to meals using a 5-point Likert scale for frequency and a 4-point Likert scale for severity

e.g. for heartburn.

Do you have any episodes of burning pain in the chest or abdomen?.

1 no.

2 once a month.

3 two to three times a week.

4 once a week.

5 daily.

How much does this problem bother you?.

1 not at all.

2 mild discomfort, yet does not interfere with day-to-day activities.

3 moderate discomfort, interferes with day-to-day activities at least once a week.

4 severe discomfort, interferes with day-to-day activities and sleep more than once a week.

#### Assessment of reflux-associated respiratory symptoms

The presence or absence of cough, shortness of breath, wheeze, and use of reliever medication at the time of experiencing each of the seven GORD symptoms was recorded (table 2).

**Table 2:** Presence of reflux-associated respiratory symptoms

**Please answer the following questions**:
• When you experience this burning pain in the chest or abdomen:
1 Do you get a cough? 2 Do you get a wheeze? 3 Do you get increased shortness of breath? 4 Do you get chest tightness? 5 Do you need to use your inhaler?
• When you experience these episodes of bitter or sour fluid or food coming back to your mouth:
1 Do you get a cough? 2 Do you get a wheeze? 3 Do you get increased shortness of breath? 4 Do you get chest tightness? 5 Do you need to use your inhaler?
• When you experience any other type of upper abdominal pain or chest pain other than the burning pain in the chest or abdomen:
1 Do you get a cough? 2 Do you get a wheeze? 3 Do you get increased shortness of breath? 4 Do you get chest tightness? 5 Do you need to use your inhaler?
• When you experience fullness in your upper abdomen :
1 Do you get a cough? 2 Do you get a wheeze? 3 Do you get increased shortness of breath? 4 Do you get chest tightness? 5 Do you need to use your inhaler?
• When you experience difficulty in swallowing:
1 Do you get a cough? 2 Do you get a wheeze? 3 Do you get increased shortness of breath? 4 Do you get chest tightness? 5 Do you need to use your inhaler?
• When you experience air coming up into your mouth [belching], after meals or not associated with meals:
1 Do you get a cough? 2 Do you get a wheeze? 3 Do you get increased shortness of breath? 4 Do you get chest tightness? 5 Do you need to use your inhaler?

Further questions were also included on smoking and alcohol habit (whether they were current or past users or had never used), on current asthma medication and duration of use. They were specifically asked whether they were taking oral salbutamol, inhaled salbutamol, oral theophylline, oral steroids and inhaled steroids as these drugs are known to induce GOR.

### Statistical analysis

Sample size estimates were made using EpiInfo (EpiInfo 6, version 6.04 (1996), Centres of Disease Control and Prevention, Atlanta, Georgia, USA and World Health Organization, Geneva, Switzerland) and other statistical analyses by SPSS for Windows (version 10, SPSS Inc., Chicago, Illinois, USA). Descriptive statistics are reported as mean and SD. Differences between groups were compared by the student t-test. Association between categorical variables was tested using Fisher Exact Test. Symptom prevalence between asthma and control groups was compared using Pearson Chi Square test. *P *values of less than 0.05 were considered significant. Cross sectional associations between GORD symptom status and whether asthmatic or not was analyzed by logistic regression analysis where GORD symptom status served as the dependent variable. Analysis was adjusted for known confounders, namely age (categorized into 10 year intervals), gender, BMI (categorized as <25 kg/m^2 ^and ≥ 25 kg/m^2^), smoking (categorized as never smoked or ever smoked) and alcohol habits (categorized as never drank or ever drank). In the asthmatic population, logistic regression was used to examine the relationship between asthma severity and presence of GORD symptoms. The analysis was adjusted for the known confounders and also for types of asthma medication whose use was found to be significantly associated with GORD symptoms on univariate analysis. Odds ratios (OR) and 95% confidence intervals (95% CI) are reported for all logistic regression analyses.

## Results

Two hundred and two asthmatics and 202 controls completed the study. They were comparable in age and sex. Demographic data of the subjects is given in tables 3 and 4.

**Table 3:** Demographic variables of subjects

	*Control* *(n = 202)*	*Asthmatic* *(n = 202)*	*P value*
Age, yrs mean (SD)	32.8 (10.2)	34.9 (11.6)	0.057*

Males, no (%)	86 (44%)	89 (44%)	0.841^†^

BMI, kg/m^2 ^mean (SD)	22.8 (2.84)	22.1 (3.03)	0.020*
Current smokers (%)	3.4	12.4	0.001^‡^
Current drinkers (%)	7.9	14.8	<0.001^‡^

* student t-test ^† ^Fisher exact test ^‡ ^Chi squared test

**Table 4:** Demographic details of asthma patients

	*Asthmatics with GORD score* *> 12.5 (n = 120)*	*Asthmatics with GORD score* *<12.5 (n = 82)*	*P value*
Age, yrs; mean (SD)	34.4 (11.4)	35.6 (11.8)	0.318

Males/females	53/67	36/46	1.000

BMI, kg/m^2^; mean (SD)	21.9 (3.3)	22.6 (2.5)	0.126

Current smoker (% of subjects)	13.3	11.0	0.630

Current drinker (% of subjects)	20.0	7.3	0.040*

Asthma severity (% of subjects)			
Mild intermittent	66.7	89.0	0.001
Mild persistent	22.5	9.8	
Moderate or severe persistent	10.8	1.2	

Asthma medication (% of subjects)			
Oral salbutamol	68.3	50.0	0.009*
Inhaled salbutamol	53.3	62.2	0.212
Oral theophylline	33.3	28.0	0.426
Oral steroids	18.3	9.8	0.092
Inhaled steroids	40.0	34.1	0.399

* Chi squared test

### Prevalence of GOR symptoms

**Figure 1:**
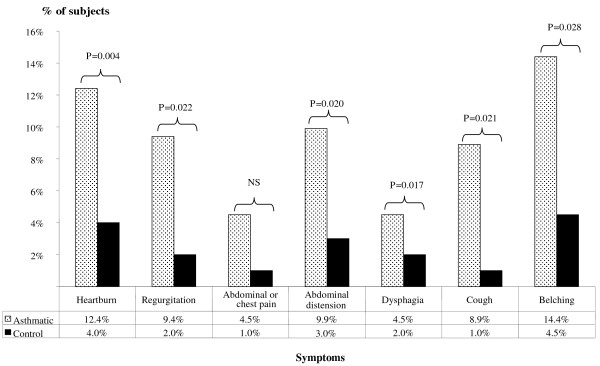
**Comparison of daily symptom frequencies in asthmatics versus controls; All comparisons using z test for comparison of proportions**.

The mean (SD) composite GORD score of asthmatics was significantly higher than that of controls (21.8 (17.2) versus 12.0 (7.6); *P *< 0.001 Mann Whitney U test). The frequency distribution of each symptom was found to be higher in asthmatics compared to controls (Figure 1). Using a cut-off of >12.5, the prevalence of symptomatic GORD in asthmatics was significantly higher than that of the controls (59.4% [95% CI, 59.1%-59.6%] versus 28.5% [95% CI, 29.0% - 29.4%]; *P *< 0.001).

### Associations between asthma and GORD symptom status

Asthmatics had a higher risk of being GORD symptom positive (score of >12.5) (OR, 3.5 [95% CI, 2.5-5.3]) and this association remained after adjustment for known confounders, namely age, gender, body mass index (BMI), alcohol and smoking status (OR, 3.7 [95% CI, 2.4 - 5.8]).

### Reflux-associated respiratory symptoms (RARS)

The total number of RARS was higher in asthmatics compared to non-asthmatics with GORD symptom scores > 12.5 (80% versus 20%, *P *< 0.05, z test) and also in asthmatics with GORD symptom scores > 12.5 compared to those with GORD symptom scores <12.5 (96% versus 4%, *P *< 0.05, z test). The commonest symptom was reflux - associated difficulty in breathing. Table 5 shows the distribution of RARS.

**Table 5:** Frequency distribution of individual Reflux-Associated Respiratory Symptoms (RARS) in subjects.

*RARS*	*Asthmatics with GORD score* *> 12.5 (n = 120)*	*Asthmatics with GORDscore* *<12.5 (n = 82)*
Heartburn associated		
*cough*	3 (2.5)	1 (1.2)
*wheeze*	4 (3.3)	-
*difficulty in breathing*	**50 (41.7)**	-
*chest tightness*	4 (3.3)	-
*inhaler use*	1 (0.8)	-

Regurgitation associated		
*cough*	2 (1.7)	1 (1.2)
*wheeze*	7 (5.8)	-
*difficulty in breathing*	**29 (24.2)**	-
*chest tightness*	2 (1.7)	-
*inhaler use*	3 (2.5)	-

Abdominal/chest pain associated		
*cough*	2 (1.7)	-
*wheeze*	2 (1.7)	-
*difficulty in breathing*	**20 (16.7)**	1 (1.2)
*chest tightness*	7 (5.8)	-
*inhaler use*	2 (1.7)	-

Abdominal distension associated		
*cough*	1 (0.8)	-
*wheeze*	3 (2.5)	1 (1.2)
*difficulty in breathing*	**16 (13.3)**	2 (2.4)
*chest tightness*	7 (5.8)	-
*inhaler use*	3 (2.5)	1 (1.2)

Dysphagia associated		
*cough*	1 (0.8)	-
*wheeze*	-	-
*difficulty in breathing*	**4 (3.3)**	-
*chest tightness*	-	-
*inhaler use*	-	-

Belching associated		
*cough*	2 (1.7)	-
*wheeze*	2 (1.7)	-
*difficulty in breathing*	**11 (9.2)**	1 (1.2)
*chest tightness*	12 (10)	1 (1.2)
*inhaler use*	3 (2.5)	-

* All values are given as number (percentage)

### Association of asthma severity and asthma medication use with GORD symptom status

One hundred and twenty asthmatics (59.4%) had a GORD symptom score >12.5 and 36% of all asthmatics experienced one or more RARS. They differed significantly from those with GORD symptom scores < 12.5 with respect to alcohol consumption, asthma severity and use of oral salbutamol but were comparable in age, gender, BMI and spirometry (Table 5). After adjusting for the five known confounding variables, multivariate analysis showed that asthma severity showed a significant dose-response association with reflux symptoms (*P *value for trend = 0.007). When compared to patients with mild intermittent asthma, those with mild persistent asthma had a higher risk (OR 2.8 (95% CI 1.1-7.0) and those with moderate or severe asthma had a much higher risk (OR 11.8 (95% CI 1.5-92.9) of being GORD symptom positive (Additional file 1, Table S1).

## Discussion

Estimates of the prevalence of GORD symptoms among adult asthmatics vary widely. The pooled sample-size weighted average prevalence of GORD symptoms in asthmatics reported in a review using data from seven cross-sectional questionnaire-based surveys was 59.2% [1]. Other individual studies have reported prevalences of 45% [2], 69.2% [6], 50% [27], 49.4% [28], 71% [29], 52.2% [30], and 51% [31]. A more limited number of studies are available from South East Asia. A prevalence of 57% in asthmatics compared to 34.1% in non-asthmatic controls has been reported from Thailand [2] and 74.8% from India [3]. Hence the prevalence of GORD symptoms seems to be similar within the South East Asian region and similar to that outside the region too. All these studies have been conducted in asthma clinics at secondary care hospitals in more urban settings [1]. As such our findings are directly comparable.

We defined GORD symptom status by use of a previously determined cut-off point on a GORD score constructed using a validated and reproducible questionnaire [26]. This composite score of GORD symptom frequency and severity has been demonstrated to have good correlation with 24 hour pH monitoring variables. We found that the mean composite GORD score was positive in 59.4% of asthmatics compared to 28.5% in non-asthmatics.

Typical symptoms such as heartburn and regurgitation are commoner than atypical symptoms in GORD patients [32,33]. From the present study, we found this to be true in our population of asthmatics. The third most frequent symptom was belching. We could not find any previous data on prevalence of belching or any other atypical symptom (chest pain or upper abdominal pain, abdominal distension, dysphagia) among asthmatics.

The correlation in the literature between asthma severity and GORD is mixed. While Field et al. [20] reported that asthma symptom severity in the week preceding their interview did not correlate with either heartburn or regurgitation, the majority of studies have shown a positive correlation [4-6,33]. The severity of asthma in our patients (as defined by ATS criteria) showed a strong and dose-dependent association with GORD symptoms, an association that was independent of known confounders for GORD. The association between asthma severity and GORD symptoms has not been described previously in the South Asian region.

As this study was a cross-sectional, there is no indication of a temporal sequence or causal relationship between these two conditions. Whether the finding that more severe forms of asthma were associated with more reflux symptoms was due to reflux aggravating asthma or severe bronchospasm promoting reflux could not be determined.

In the present study, 36% of the asthmatics experienced reflux-associated respiratory symptoms (RARS) and these occurred more frequently in subjects who had positive GORD symptom scores. Other studies have investigated the association between typical GORD symptoms and respiratory symptoms. Field et al. reported a prevalence of 41.2%. In this study 45 asthmatics out of 109 reported at least one RA0RS [20]. Another study reported that 'moderately bothersome' heartburn and acid regurgitation were significantly associated with asthma symptoms (odds ratio = 3.2; 95% confidence interval = 1.6-6.4), wheeze (OR = 3.5; 95% CI = 1.7-7.2), and nocturnal cough (OR = 4.3; 95% CI = 2.1-8.7) independently of body mass index. This is the first report of an association of RARS with atypical symptoms of GORD.

Bronchodilator medication may predispose to gastro-oesophageal reflux: theophylline has been shown to stimulate gastric acid secretion and lower LOS pressure, oral beta adrenergic agonists have been implicated in lowering the LOS pressure [10,34],  and oral corticosteroids have been shown to increase oesophageal acid contact times [11]. Debley et al. [35] demonstrated that GORD symptoms were associated with the use of asthma medication. A number of studies contradict these findings. Sontag et al. [36] showed that there was no significant difference in reflux patterns in asthmatics who were taking anti-asthmatic drugs versus those not taking them. Field et al. [20] noted that none of the asthma medications were associated with an increased likelihood of having symptoms of GORD. The Nord-Trondelag health survey showed that asthmatics had reflux symptoms to a 60% greater extent than non-asthmatics. However, asthma nor the use of asthma medication influenced the risk of GORD [37]. We found that 64.4% of our asthmatics used one more oral drugs (beta_2 _agonists, xanthines or steroids) for control of asthma. Asthmatics with positive GORD symptoms scores had significantly higher usage of oral salbutamol on univariate analysis. However adjustment for oral salbutamol use alone, and subsequently for all five asthma medications did not substantially alter the risk estimates for the different categories of asthma severity. Thus we have demonstrated that use of asthma medication, oral or otherwise, does not seem to influence the presence or absence of GORD. This has not been previously reported in an Asian population.

Several factors are known to contribute to both GORD and asthma. To reduce the risk of confounding factors we adjusted statistically for all plausible confounders. Alcohol consumption and smoking are known to be associated with GORD [38]. Recent reports indicate that in Sri Lanka, 41% of males and 3.4% of females are current smokers and 23% of men and 0.9% of women take alcohol at least monthly [39]. In our study, the percentage of current smokers (20%) and drinkers (13.3%) was lower than this. This could have been because these patients were hospital clinic attendees who are regularly exposed to health advice. The low prevalence may have resulted in our failing to show an influence of these factors on the presence of GORD symptoms in asthmatics, though it may also represent something specific about asthmatics and GORD and warrant further investigation. Increased body weight [40], increasing age and male sex [41] are also known to be associated with GORD. Our study design was such that asthmatics and controls were comparable in age and sex. An asthmatic had a higher risk of being GORD symptom positive compared to a non-asthmatic even after adjustment for known confounders. This indicates a lack of strong confounding by these factors, even though there was a significant difference in BMI, alcohol and tobacco consumption among the two groups. These findings are in agreement with previous studies [36].

## Conclusion

In summary, use of an interviewer administered GORD questionnaire has shown a higher prevalence of GORD symptoms and RARS in a cohort of adult Sri Lankan asthmatics compared to non-asthmatics. The use of asthma medication does not influence the presence or absence of GORD though asthma severity shows a significant association. There is a significant burden of RARS morbidity in the region. We have now shown that the prevalence of RARS is associated not only with typical symptoms but also with atypical symptoms of GORD. This suggests that reflux associated respiratory symptoms may benefit from more rigorous investigation and, even when typical symptoms are absent, treatment of GORD may be indicated.

## Competing interests

The authors declare that they have no competing interests.

## Authors' contributions

All authors read and approved the final manuscript. All authors were involved in study conception, design, manuscript drafting and revision. In addition, LDA and AP were involved in statistical analysis. In addition, LDA was involved in data acquisition.

## Authors' information

LDA: MBBS, Lecturer, Department of Physiology, Faculty of Medicine, University of Kelaniya, Ragama, Sri Lanka. AP : MBBS, MD, Professor of Public Health, Department of Public Health, Faculty of Medicine, University of Kelaniya, Ragama, Sri Lanka. HJDS : MBBS, MD, FRCP, FCCP, D Phil (Oxon), Senior Professor of Medicine, Department of Medicine, Faculty of Medicine, University of Kelaniya, Ragama, Sri Lanka. CDR : MBBS, BSc, MRCP, DTM&H (Lond), Senior Lecturer in Pharmacology, Department of Pharmacology, Faculty of Medicine, University of Kelaniya, Ragama, Sri Lanka.

## Pre-publication history

The pre-publication history for this paper can be accessed here:

http://www.biomedcentral.com/1471-2466/10/49/prepub

## Supplementary Material

Additional file 1 **Table S1 - Asthma severity, asthma medication use and GORD symptom status**. Click here for file
