# Supplementary material for: Prevalence of gastro-oesophageal reflux disease symptoms and reflux-associated respiratory symptoms in asthma
Source: BMC Pulm Med. 2010 Sep 15;10:49. doi: 10.1186/1471-2466-10-49 (PMC2954896; doi:10.1186/1471-2466-10-49)
Supplement: Additional file 1 — Table S1 - Asthma severity, asthma medication use and GORD symptom status. [file 1471-2466-10-49-S1.DOC]

**Table S1 - Asthma severity, asthma medication use and GORD symptom status**

| ***Variables*** | ***Asthmatics with GORD score>12.5 (n=120)*** | ***Asthmatics with GORD score<12.5(n=82)*** | ***Unadjusted model***  ***OR 95% CI*** | ***P value*** | ***Multivariable model^†^***  ***OR 95% CI*** | ***P value*** | |
| --- | --- | --- | --- | --- | --- | --- | --- |
| Asthma severity  Mild, intermittent  Mild, persistent  Moderate/ severe persistent | 80 (66.7%)  27 (22.5%)  13 (10.8%) | 73 (89.0%)  8 (9.8%)  1 (1.2%) | 1.0 reference  3.08 1.3-7.2  11.8 1.5-92.9 | 0.003 | 1.0 reference   - 1. 1.1-7.0   11.8 1.4-94.4 | | 0.023 |
| Use of oral salbutamol  No  Yes | 38 (31.7%)  82 (68.3%) | 41 (50.0%)  41 (50.0%) | 1.0 reference  2.1 1.2-3.8 | 0.009 | 1.0 reference  1.8 0.8-4.0 | | 0.158 |
| Use of inhaled salbutamol  No  Yes | 56 (46.7%)  64 (53.3%) | 31 (37.8%)  51 (62.2%) | 1.0 reference  0.6 0.4-1.2 | 0.212 | 1.0 reference  1.0 0.4-2.3 | | 0.959 |
| Use of oral theophylline  No  Yes | 80 (66.7%)  40 (33.3%) | 59 (72.0%)  23 (28.0%) | 1.0 reference  1.2 0.7-2.3 | 0.426 | 1.0 reference  0.9 0.4-2.2 | | 0.905 |
| Use of oral steroids  No  Yes | 98 (81.7%)  22 (18.3%) | 74 (90.2%)  8 (9.8%) | 1.0 reference  2.0 0.8-4.9 | 0.097 | 1.0 reference  2.0 0.7-5.5 | | 0.193 |
| Use of inhaled steroids  No  Yes | 72 (60.0%)  48 (40.0%) | 54 (65.9%)  28 (34.1%) | 1.0 reference  1.2 0.7-2.3 | 0.399 | 1.0 reference  1.0 0.5-2.1 | | 0.799 |

* Data are presented as No. (%). ^†^ In the multivariable logistic regression model, GORD symptom status serves as the dependent variable and all analyses are adjusted for the confounding variables age, gender, BMI, smoking and alcohol status. OR = odds ratio. 95% CI = 95% confidence intervals.
